# Pathways from maternal depression to young adult offspring depression: an exploratory longitudinal mediation analysis

**DOI:** 10.1002/mpr.1520

**Published:** 2016-07-29

**Authors:** Artemis Koukounari, Argyris Stringaris, Barbara Maughan

**Affiliations:** ^1^ Department of Biostatistics, Institute of Psychiatry, Psychology and Neuroscience King's College London London UK; ^2^ Department of Child and Adolescent Psychiatry, Institute of Psychiatry, Psychology and Neuroscience King's College London London UK; ^3^ MRC Social, Genetic and Developmental Psychiatry Centre, Institute of Psychiatry, Psychology and Neuroscience King's College London London UK

**Keywords:** ALSPAC, maternal depression, young adult depression, emotional and conduct problems, longitudinal mediation

## Abstract

Maternal depression in the peri‐natal period is associated with increased risk for young adult depression in offspring. This study explored mediation of these links via trajectories of child conduct and emotional problems (Strengths and Difficulties Questionnaire) from ages 4–16 years old in data from the Avon Longitudinal Study of Parents and Children cohort (*n* = 13373). Through gender‐specific structural equation models, a composite measure of *exposure* to early maternal depression (Edinburgh Postnatal Depression Scale), predicted young adult depression at age 18 (Revised Clinical Interview Schedule – *distal outcome*). Mediational effects were then estimated by testing which parts of joint piecewise latent trajectory models for child/adolescent conduct and emotional problems were associated with both exposure and distal outcome. For girls, only conduct problems in early childhood were consistently indicated to mediate effects of early maternal depression on risk of young adulthood depression. Some evidence for a pathway via changing levels of childhood and adolescent emotional difficulties was also suggested. For boys, by contrast, the differing models gave less consistent findings providing some evidence for a small time‐specific indirect effect via early childhood conduct problems. In addition to its practice implications the current methodological application offers considerable potential in exploratory longitudinal developmental mediation studies. © 2016 The Authors International Journal of Methods in Psychiatric Research Published by John Wiley & Sons Ltd

## Introduction

The life course approach to epidemiology has been crucial in highlighting associations between pre‐natal and early post‐natal exposures and offspring outcomes much later in development (Kuh *et al*., 2003; Lynch and Smith, 2005; Pickles *et al*., 2007). The next key step – essential for both theoretical advance and clinical utility – is to trace the pathways by which such links are mediated.

We focus here on one well‐documented association of this kind: links between pre‐/early post‐natal depression in mothers and risk of depression in their offspring. Increased rates of childhood emotional/behavioural difficulties in the offspring of depressed mothers have been documented for many years (Stein *et al*., 2014). More recently, maternal depression has also been linked with elevated rates of emotional/behavioural difficulties (Pearson *et al*., 2013) and increased risk of depression diagnoses (Plant *et al*., 2015), in offspring in adult life (Betts *et al*., 2015). Given the heavy burden of disability associated with depression (Collins *et al*., 2011; Ferrari *et al*., 2013) these long‐term linkages raise issues of major scientific and public health concern.

Mediation of the intergenerational transmission of risk for depression is likely to run through a variety of pathways; to date, genetic and epigenetic effects, other biologically‐based influences, the impact of maternal depression on parenting, and associations of parental depression with other adverse childhood exposures have all been implicated in different samples (Stein *et al*., 2014). In this paper we explore the role of child characteristics, and in particular emotional and behavioural difficulties in childhood. Early emotional and behavioural problems are a strong candidate as mediators of risk for depression: both are established sequelae of maternal depression (Barker *et al*., 2012), and both (emotional problems via homotypic continuities, and behavioural problems via widely replicated patterns of heterotypic continuity) are associated with increased risk for depression later in life (Maughan *et al*., 2013). Importantly, both are also readily identifiable, and potentially modifiable, targets for intervention. Examining their roles in the context of coherent developmental models, however, poses key methodological challenges. First, emotional and behavioural problems co‐occur across childhood and adolescence (Barker *et al*., 2010), and variations in their patterns of co‐development, as well as in trajectories of specific difficulties, may be important for later outcomes. Second, though emotional and behavioural difficulties can be evident from early childhood, little is known about the ages at which they may be most salient, or the implications of the marked rise that both sets of difficulties typically show in early adolescence (Ferrari *et al*., 2013; Maughan *et al*., 2013). Finally, well‐established gender differences in *rates* of such problems (conduct problems more common in boys, emotional difficulties more common in girls) may signal related differences in intervening processes. Models that account for these various complexities, as well as exploring potential gender differences in mechanisms, are essential to extend our understanding of *when* and for *whom* key mediating effects might occur.

So far as we are aware no studies have addressed this full range of issues thus far. Nilsen *et al*. (2013) used structural equation modelling to examine associations among maternal distress and child internalizing and externalizing problems from early childhood (child age 1.5 years) to early adolescence (age 12.5), and to explore the roles of all of these factors as predictors of offspring depression at ages 14.5 and 16.5 years. Maternal distress was associated with increased risks for child internalizing and externalizing problems, but showed no direct links with adolescent depression. Instead, indirect pathways to depression were mediated via prior emotional and behavioural difficulties, with externalizing problems assessed as early as 4.5 years showing direct associations with risk for depression in the mid‐teens. Although most associations were similar for boys and girls, some gender‐specific effects were also identified. Pointers to the impact of developmental change in levels of conduct problems can be gleaned from studies using latent class growth analyses to identify differing developmental trajectories of child and adolescent conduct problems. Here, current findings are inconclusive; our own past study in the cohort reported on here (Stringaris *et al*., 2014) suggested that ‘adolescent onset’ as well as early onset persistent conduct problems were associated with increased risk for depression in early adulthood, while Odgers *et al*. (2008) found no comparable effects for depression assessed in the early thirties.

We now extend and elaborate on these findings, using data from the Avon Longitudinal Study of Parents and Children (ALSPAC) to develop longitudinal structural equation models (SEMs) to incorporate each of the requirements outlined earlier. Building on our prior findings of links between childhood conduct problems and early adult depression in this cohort (Stringaris *et al*., 2014), we begin by testing the extent to which trajectories of disruptive behaviour problems across childhood and adolescence mediate links between maternal depression in pregnancy and the early post‐natal period and offspring depression in early adult life. Next, we re‐evaluate these findings including parallel trajectories of emotional difficulties. In addition to illuminating these specific substantive issues, we hope our study will serve as an illustrative example to guide others in the application of complex SEMs for investigating longitudinal mediation and generating hypotheses where a broad range of effects are potentially of interest (VanderWeele, 2012).

## Methods

### Sample

ALSPAC (http://www.bris.ac.uk/alspac) is an ongoing population‐based study designed to investigate the effects of a wide range of factors on health and development. All women resident in Avon, UK with expected dates of delivery between 1 April 1991 and 31 December 1992 were eligible for participation. The resulting study cohort consisted of 14,541 pregnancies and 13,988 children still alive at 12 months of age. Compared to the 1991 UK National Census Data, the sample showed a slightly higher proportion of house owner‐occupiers and a smaller proportion of mothers from ethnic minorities (Boyd *et al*., 2013). Ethical approval for the study was obtained from the ALSPAC Law and Ethics Committee and local Research Ethics Committees. Please note that the study website contains details of all the data that is available through a fully searchable data dictionary: http://www.bris.ac.uk/alspac/researchers/data-access/data-dictionary.

### Measures

#### Maternal pre‐ and post‐natal depression

Symptoms of maternal depression were measured using the Edinburgh Post‐natal Depression Scale (EPDS) (Cox *et al*., 1987) at approximately 18 and 32 weeks ante‐natally and eight weeks and eight months post‐natally. The EPDS is a 10 item self‐report questionnaire specifically designed to screen for peri‐natal depression by avoiding using physical symptoms which may lead to measurement error in this period. Several studies have reported Cronbach's *α* > 0.75 and strong validity, recommending EPDS as a useful means of detecting women at risk of post‐natal depression (Teissèdre and Chabrol, 2004).

#### Child and adolescent conduct and emotional problems

Maternal reports of child conduct and emotional problems were collected at ages 4, 7, 8, 10, 12, 13 and 16 years using the Strengths and Difficulties Questionnaire (SDQ), a widely‐used screening instrument with well‐established reliability (mean Cronbach's *α* = 0.73) and validity as judged against psychiatric diagnoses (Goodman, 1997, 2001). The conduct problem subscale of the SDQ includes five items indexing fighting, lying, stealing, disobedience and temper outbursts. The emotional problems subscale includes five items assessing worries, fears, somatic symptoms and unhappiness.

#### Young adult depression

Young adult depression was assessed using the Revised Clinical Interview Schedule [CIS‐R] (Lewis *et al*., 1992), a self‐administered, computerized interview completed at age 18 at a research clinic (mean age at attendance 17 years 10 months). The CIS‐R establishes the severity of core symptoms of depressive disorders (depression, depressive thoughts, fatigue, sleep and concentration problems). Each symptom is scored on a 0–4 scale (depressive thoughts 0–5), according to the severity (frequency, duration and unpleasantness) of the symptom experienced (Cronbach's *α* = 0.77 in this cohort (Stringaris *et al*., 2014)). We used total symptom scores as the dependent variable in the analyses.

### Statistical analysis

We began by examining gender‐specific correlations for all exposure, mediator and dependent variables, and gender‐specific mean scores for conduct and emotional problems at each measurement point in childhood and adolescence, and for depression symptoms at age 18. We also generated plots from ordinary least squares (OLS)‐estimated individual growth trajectories using the SAS‐based OLStraj macro (Carrig *et al*., 2004) for conduct and emotional problems before any statistical modelling took place (data not shown – but available from authors upon request).

Having tested the measurement models first and typically one at a time we then moved to include and test the structural parts. We specifically fitted gender‐specific SEMs in Mplus version 7.3 (Muthén and Muthén, 1998–2012), using full information maximum likelihood estimation and exploiting all available data (*n* = 6917 for boys and *n* = 6456 for girls) – including those partially missing – with the aim of providing consistent estimates under the assumption of data missing at random (Little and Rubin, 2002). There were 994 (14.37%) boys and 1152 (17.84%) girls with complete data on all the measures examined. Furthermore, 46.71% and 46.70% of offspring boys as well as 49.92% and 49.85% of offspring girls had at least five out of seven complete data points on conduct and emotional problems, respectively.

The final models were selected based on examination of root mean square error of approximation (RMSEA), accompanied by its associated 90% confidence interval (CI), the comparative fit index (CFI), likelihood ratio tests between more and less restrictive nested models, and the Bayesian Information Criterion (BIC).

#### Factor model for maternal depression

Both pre‐ and post‐natal maternal depression have been associated with increased risk for young adult depression in offspring in this cohort (Pearson *et al*., 2013). We thus used a composite measure of exposure to maternal depression at both time‐periods, modelled as a latent variable *U* through a confirmatory factor analysis (CFA) to account for the variation and co‐variation of the four selected observed EPDS scores (De Stavola *et al*., 2006). The measurement part of the model because *U* is not observed but is proxied by *EPDS*_1_, *EPDS*_2_, *EPDS*_3_ and *EPDS*_4_ is defined as

$$\begin{array}{l} E\left({\text{EPDS}}_{1}\right)=\mu_{1}+\lambda_{1}U\\ \begin{array}{l} \begin{array}{l} E\left(EPDS_{2}\right)\;=\mu_{2}+\lambda_{2}U\\ E\left(EPDS_{4}\right)\;=\mu_{4}+\lambda_{4}U \end{array}\\ E\left(EPDS_{3}\right)\;=\mu_{3}+\lambda_{3}U \end{array} \end{array}$$

where *E*(…) stands for expectation and the latent variable *U* as well as its proxy variables are assumed to be normally distributed with the parameters *μ*_1,_ *μ*_2,_ *μ*_3_ and *μ*_4_ to be set to zero. Through this model and given *U*, we assume that the observed variables are independent of one another and they are only related to each other through their common relationship with *U*. This approach yielded acceptable fit of CFI = 0.97 and 0.96 and RMSEA = 0.12 (90% CI = 0.11–0.14) and 0.129 (90% CI = 0.12–0.14) for boys and girls, respectively.

#### Univariate piecewise latent trajectory models for conduct and emotional problems

Piecewise models allow separate slopes to be fitted to repeated observations occurring before and after a “critical period” or “event” capturing non‐linearity through the use of additional latent growth factors (Bollen and Curran, 2006; Duncan *et al*., 2011). In this study we considered univariate two‐piece linear models, which have two linear latent slope factors, *β*_1*i*_ and *β*_2*i*_, to describe two “pieces” of linear change occurring over two separate segments of time. In scalar terms, the univariate model is

$$E\left(Y_{\text{it}}\right)\;=\alpha_{i}+\lambda_{1t}\beta_{1i}+\;\lambda_{2t}\beta_{2i}$$

where, *Y_it_* is the observed value of repeated measure *Y* (e.g. conduct or emotional problems) for individual *i* at time point *t*, *α_i_* is a latent intercept variable at the initial SDQ assessment period and unique to each individual in the sample, *λ_t_* = 0, 0.3, 0.4, 0.8, 1 and 1.2 reflects unequally spaced linear change as a function of SDQ age assessment period (Biesanz *et al*., 2004; Bollen and Curran, 2006; Curran and Willoughby, 2003) and each latent slope factor is further described in terms of a mean (*μ_α_*, *μ*_*β*1_ and *μ*_*β*2_) and variance (*ζ_α_*, *ζ*_*β*1*i*_ and *ζ*_*β*2*i*_). The values of the intercept *α_i_* and the linear latent slope factors, *β*_1*i*_ and *β*_2*i*_ are contingent on how *λ_t_* is coded. Initial investigation of the growth univariate trajectories of conduct and emotional problems in the current data‐set, along with developmental theory (Moffitt, 1993; Thapar *et al*., 2012) led us to place the knot (i.e. the transition point from one piece of the trajectory to the next) at age 10 for all individuals in the population (Flora, 2008) for both sets of difficulties. In contrast with more traditional autoregressive (AR) cross‐lagged panel models, which only capture inter‐individual change over time, piecewise models consider both the between‐wave covariance matrix and the observed mean structure.

#### SEM approach: combined factor model and piecewise latent trajectory models in relation to distal outcome

The main SEM analyses then proceeded in three stages. First, we examined the relationship between the factor model representing *exposure* to maternal depression with the dependent variable i.e. the observed *distal outcome* of young adult depression at age 18 (see Supporting Information Figure S1 and Model S1).

**Figure 1:**
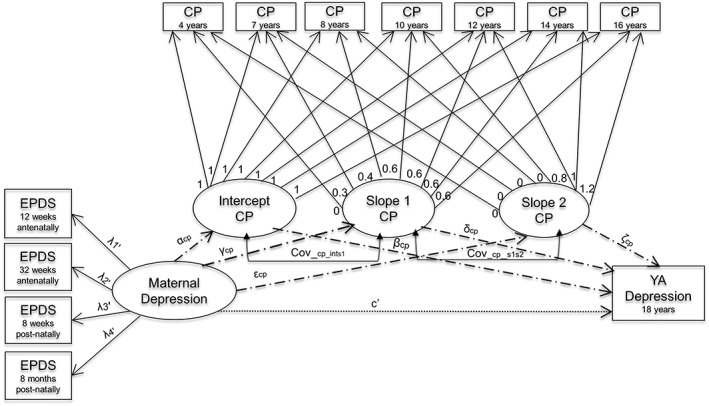
Path diagram for Model 2. Abbreviations: YA Depression 18, young adult depression, age 18; CP, observed measures of conduct problems. In this figure and all figures representing fitted SEMs, Greek letters as well as the letter “c” represent regression coefficients for the paths from the independent variable to the dependent variable. Those letters are the same as those representing the pathways indicated with arrows in the corresponding tables. Squares indicate observed variables while circles indicate latent variables. In the piecewise linear model note that for age 4, the factor loading for both slope factors is set to 0 thus establishing the status factor reference point as the predicted level of age 4. Because the first linear slope covers change from age 4–10 but not afterwards the loadings for this factor are set to be constant after the last point. The second slope factor, which covers change from age 10–16, then picks up where the first slope factor left off with non‐zero loadings for ages 10–16. Lamdas (λ) on the pathways are factor loadings from the factor model of maternal depression; those estimates are not included in the tables for simplicity but are available from authors.

**Figure 2:**
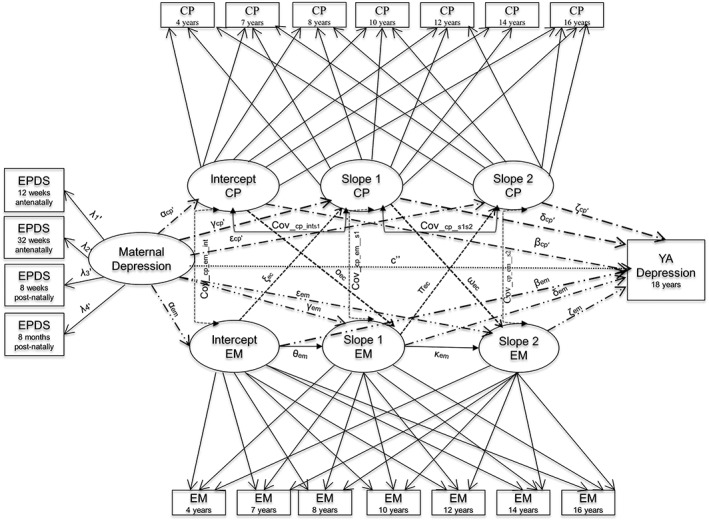
Path diagram for Model 3. Abbreviations: YA Depression 18, young adult depression, age 18; CP, observed measures of conduct problems; EM, observed measures of emotional problems. We use primes after the path coefficients to distinguish between the corresponding coefficients derived from different models.

Next we examined mediation of these links via trajectories of child conduct and emotional problems. We began by testing which parts of the univariate piecewise latent trajectories for *conduct problems* (intercepts at age four, and slopes from ages 4–10 years [slope 1] and 10–16 years [slope 2]) acted as mediators of the relationship between maternal depression and young adult depression (Figure 1 and Model 2 in Table 1). Partial (MacKinnon, 2008) or complementary (Zhao *et al*., 2010) mediation is suggested when the magnitude of the direct effect is reduced (here, comparing relevant estimates from Models 1 and 2 [*c*’ < *c*]) after the introduction of potential mediators. The product of coefficients method was used to obtain point estimates of the indirect effects for path tracing (MacKinnon and Dwyer, 1993; MacKinnon *et al*., 2002; Sobel, 1982). Bootstrap techniques based on 1000 samples were used to obtain more accurate estimates of the CIs of the indirect, as well as direct and total effects. We also report standardized regression coefficients (reflecting the change in the outcome variable per a one standard deviation (SD) change in the independent variable), allowing substantive interpretation of the results for all models (see Supporting Information Tables S3 and S4) in both genders. Next we tested the effects of including comparable indicators of *emotional problems* over the 4–16 year age‐period (Figure 2 and Model 3 in Table 1) to distinguish the direct effect of maternal depression on young adult offspring depression from the total and time‐specific indirect effects of conduct and emotional problems.

**Table 1:** Total, direct, total indirect and time‐specific indirect effects of pre‐ and post‐natal maternal depression on young adult depression with 95 % Bootstrap confidence intervals (CIs) and *p*‐values for Models 2 and 3

Model 2 Conduct problem trajectories as mediators			Model 3 Conduct and emotional problem trajectories as mediators		
	Standardized estimates (95% Bootstrap CIs)			Standardized estimates (95% Bootstrap CIs)	
Effect	Boys (*n* = 6917)	Girls (*n* =6456)	Effect	Boys (*n* = 6917)	Girls (*n* =6456)
Total^a^	0.147 (0.088 to 0.206) *p* < 0.001	0.168 (0.118 to 0.219) *p* < 0.001	Total^a^	0.155 (0.096 to 0.213) *p* < 0.001	0.171 (0.121 to 0.221) *p* < 0.001
Direct c’:^b^ Mat dep→ YA Depression 18	0.128 (0.064 to 0.192) p < 0.001	0.095 (0.036 to 0.153) *p* = 0.002	Direct c:^b^ Mat dep → YA Depression 18	0.110 (−0.047 to 0.268) *p* = 0.170	0.092 (0.017 to 0.167) *p* = 0.016
Total indirect:^c^ (α_cp_ × β_cp_) + (γ_cp_ × δ_cp_) + (ε_cp_ × ζ_cp_)	0.019 (−0.002 to 0.041) *p* = 0.082	0.074 (0.047 to 0.101) p < 0.001	Total indirect:^c^ (α_cp’_ × β_cp’_) + (γ_cp’_ × δ_cp’_) + … +(α_em_ × θ_em_ × k_em_ × ζ_em_)	0.044 (−0.102 to 0.191) *p* = 0.553	0.079 (0.028 to 0.131) *p* = 0.002
Time‐specific indirect:^d^			Time‐specific indirect:^d^		
α_cp_ × β_cp_ : Mat dep → Int_cp→ YA Depression 18	0.027 (0.006 to 0.048) *p* = 0.011	0.063 (0.041 to 0.084) p < 0.001	α_cp’_ × β_cp’_: Mat dep → Int_cp → YA Depression 18	0.013 (−0.101 to 0.127) *p* = 0.823	0.038 (0.010 to 0.066) *p* = 0.008
γ_cp_ × δ_cp_: Mat dep → S1_cp→ YA Depression 18	0.000 (−0.002 to 0.003) *p* = 0.846	−0.005 (−0.016 to 0.005) *p* = 0.328	γ_cp’_ × δ_cp’_: Mat dep → S1_cp → YA Depression 18	−0.046 (−0.239 to 0.148) *p* = 0.644	0.002 (−0.013 to 0.017) *p* = 0.795
ε_cd_ × ζ_cp_: Mat dep → S2_cp → YA Depression 18	−0.008 (−0.020 to 0.004) *p* = 0.178	0.017 (−0.002 to 0.036) *p* = 0.084	ε_cp’_ × ζ_cp’_: Mat dep → S2_cp → YA Depression 18	0.008 (−0.028 to 0.044) *p* = 0.645	0.009 (−0.015 to 0.033) *p* = 0.477
			α_em_ × β_em_: Mat dep → Int_em → YA Depression 18	−0.012 (−0.497 to 0.472) *p* = 0.960	0.008 (−0.072 to 0.088) *p* = 0.848
			γ_em_ × δ_em_: Mat dep → S1_em → YA Depression 18	0.069 (−0.112 to 0.250) *p* = 0.455	0.023 (−0.016 to 0.062) *p* = 0.241
			ε_em_ × ζ_em_: Mat_dep → S2_em → YA Depression 18	−0.003 (−0.075 to 0.069) *p* = 0.938	0.017 (−0.008 to 0.042) *p* = 0.179
			α_em_ × ξ_ec_ × δ_cp’_: Mat dep → Int_em → S1_cp → YA Depression 18	0.038 (−0.129 to 0.205) *p* = 0.657	−0.003 (−0.027 to 0.020) *p* = 0.783
			γ_em_ × π_ec_ × ζ_cp’_: Mat dep → S1_em → S2_cp → YA Depression 18	0.004 (−0.013 to 0.022) *p* = 0.632	−0.002 (−0.010 to 0.005) *p* = 0.529
			α_cp’_ × o_ec_ × δ_em_: Mat dep → Int_cp → S1_em → YA Depression 18	−0.025 (−0.244 to 0.194) *p* = 0.822	0.001 (−0.036 to 0.039) *p* = 0.953
			α_em_ × θ_em_ × δ_em_: Mat dep → Int_em→ S1_em → YA Depression 18	0.053 (−0.478 to 0.583) *p* = 0.846	0.001 (−0.087 to 0.088) *p* = 0.990
			γ_cpʹ_ × ω_ec_ × ζ_em_: Mat dep → S1_cp → S2_em → YA Depression 18	0.018 (−0.046 to 0.081) *p* = 0.585	0.001 (−0.002 to 0.004) *p* = 0.706
			γ_em_ × k_em_ × ζ_em_: Mat dep → S1_em → S2_em → YA Depression 18	−0.042 (−0.127 to 0.042) *p* = 0.327	−0.012 (−0.030 to 0.007) *p* = 0.206
			α_cp’_ × o_ec_ × π_ec_ × ζ_cd’_: Mat dep → Int_cp → S1_em → S2_cp → YA Depression 18	−0.002 (−0.018 to 0.015) *p* = 0.855	0.000 (−0.004 to 0.003) *p* = 0.949
			α_em_ × θ_em_ × π_ec_ × ζ_cp’_: Mat dep → Int_em → S1_em → S2_cp → YA Depression 18	0.003 (−0.038 to 0.044) *p* = 0.877	0.000 (−0.008 to 0.008) *p* = 0.988
			α_em_ × ξ_ec_ × ω_ec_ × ζ_em_: Mat dep → Int_em → S1_cp → S2_em → YA Depression 18	−0.015 (−0.065 to 0.036) *p* = 0.568	−0.001 (−0.005 to 0.003) *p* = 0.651
			α_cp’_ × o_ec_ × k_em_ × ζ_em_: Mat dep → Int_cp → S1_em → S2_em → YA Depression 18	0.015 (−0.058 to 0.089) *p* = 0.680	−0.001 (−0.015 to 0.014) *p* = 0.937
			α_em_ × θ_em_ × k_em_ × ζ_em_: Mat dep → Int_em → S1_em → S2_em → YA Depression 18	−0.032 (−0.207 to 0.142) *p* = 0.716	0.000 (−0.033 to 0.033) *p* = 0.987
Model fit information					
RMSEA (90 % CI)	0.040 (0.037 to 0.043)	0.038 (0.035 to 0.041)	RMSEA (90 % CI)	0.038 (0.036 to 0.040)	0.036 (0.035 to 0.038)
CFI	0.970	0.972	CFI	0.952	0.955
BIC	227260.597	218842.292	BIC	317711.561	309925.118
Log‐likelihood	−113471.147	−109263.236	Log‐likelihood	−158572.85	−154681.831
Number of parameters	36	36	Number of parameters	64	64

Abbreviations: Mat dep, Maternal depression; YA Depression 18, young adult depression, age 18; Int__cp_, Intercept for conduct problems, age 4; S1__cp_, Slope 1 for conduct problems‐change ages 4–10 years; S2__cp_, Slope 2 for conduct problems‐change ages 10–16 years; Int__em_, Intercept for emotional problems, age 4; S1__em_, Slope 1 for emotional problems‐change ages 4–10 years; S2__em_, Slope 2 for emotional problems‐change ages 10–16 years; RMSEA, root mean square error of approximation; 90% confidence interval (CI); CFI, comparative fit index; BIC, Bayesian Information Criterion.

a Total effect: the sum of all effects (i.e. direct and indirect) of pre‐ and post‐natal maternal depression on young adult depression at age 18.

b Direct effect: unmediated effect of pre‐ and post‐natal maternal depression on young adult depression at age 18.

c Total indirect effect: degree to which assumed mediators (i.e. initial levels of conduct problems at age 4 and changes of conduct problems during ages 4–10 and 10–16 for Model 2; initial levels of conduct problems and emotional problems at age 4 and changes of conduct and emotional problems during ages 4–10 and 10–16 for Model 3) mediate the pre‐ and post‐natal maternal depression to young adult depression at 18. The overall indirect effect consists of the sum of all time‐specific indirect effects of pre‐ and post‐natal maternal depression on young adult depression at 18.

d Time‐specific indirect effects: degree to which assumed mediators at specific time points (ages) mediate the effect of pre‐ and post‐natal maternal depression on young adult depression at age 18.

Finally, we fitted a reduced model in which all non‐significant paths were constrained to zero testing the assumption of conditional independence between remaining variables (Model 4, Table 2 and Supporting Information Figures S2 and S3).

**Table 2:** Total, direct, total indirect and time‐specific indirect effects of maternal depression on young adult depression with 95% Bootstrap confidence intervals (CIs) and *p*‐values for Model 4

Model 4^1^			
Effect	Standardized estimates (95% Bootstrap CIs)	Effect	Standardized estimates (95% Bootstrap CIs)
Boys (*n* = 6917)		Girls (*n* = 6456)	
Total	0.154 (0.095 to 0.213) *p* < 0.001	Total	0.172 (0.122 to 0.221) *p* < 0.001
Direct c”: Mat dep → YA Depression18	0.149 (0.091 to 0.208) *p* < 0.001	Direct c”: Mat dep → YA Depression 18	0.102 (0.046 to 0.158) *p* < 0.001
Total indirect	0.004 (−0.003 to 0.012) *p* = 0.266	Total indirect	0.070 (0.043 to 0.097) *p* < 0.001
Time‐specific indirect		Time‐specific indirect	
γ_cp”_ × δ_cp”_: Mat dep → S1_cp → YA Depression 18	−0.018 (−0.051 to 0.016) *p* = 0.305	α_cp”_ × β_cp”_: Mat dep → Int_cp → YA Depression 18	0.037 (0.017 to 0.056) *p* < 0.001
α_em’_ × ξ_ec’_ × δ_cp’_: Mat dep → Int_em → S1_cp YA Depression 18	0.025 (−0.012 to 0.061) *p* = 0.189	γ_em”_ × δ_em”_: Mat dep → S1_em → YA Depression 18	0.028 (0.007 to 0.048) *p* = 0.009
γ_cd”_ × ω_ec”_ × ζ_em”_: Mat dep → S1_cp → S2_em → YA Depression 18	0.006 (−0.015 to 0.028) *p* = 0.563	ε_em”_ × ζ_em”_: Mat_dep → S2_em → YA Depression 18	0.020 (0.002 to 0.040) *p* = 0.030
α_em’_ × ξ_ec’_ × ω_ec’_ × ζ_em’_: Mat dep → Int_em → S1_cp → S2_em YA Depression 18	−0.009 (−0.034 to 0.016) *p* = 0.490	γ_em”_ × k_em”_ × ζ_em”_: Mat dep → S1_ em → S2_em → YA Depression 18	−0.016 (−0.028 to −0.004) *p* = 0.013
Model fit information			
RMSEA (90 % CI)	0.037 (0.036 to 0.039)	RMSEA (90 % CI)	0.035 (0.034 to 0.037)
CFI	0.950	CFI	0.955
BIC	317680.134	BIC	309869.563
Log‐likelihood	−158592.5	Log‐likelihood	−154684.8
Number of parameters	56	Number of parameters	57

Abbreviations: Mat dep, Maternal depression; YA Depression 18, young adult depression, age 18; Int__cp_, Intercept for conduct problems, age 4; S1__cp_, Slope 1 for conduct problems‐change ages 4–10 years; S2__cp_, Slope 2 for conduct problems‐change ages 10–16 years; Int__em_, Intercept for emotional problems, age 4; S1__em_, Slope 1 for emotional problems‐change ages 4–10 years; S2__em_, Slope 2 for emotional problems‐change ages 10–16 years; RMSEA, root mean square error of approximation; 90% confidence interval (CI); CFI, comparative fit; BIC, Bayesian Information Criterion.

Model 4 contains the same pathways as Model 3 but omitting those which were not significant in the latter for each gender separately. For boys, the omitted pathways from Model 3 in Model 4 were as follows: β_cp_’, ε_cd_’, ζ_cd_’, β_em_, γ_em_, θ_em_ and o_ec_. For girls the omitted pathways from Model 3 in Model 4 were as follows: γ_cp_’, δ_cp_’, ζ_cp_’, β_em_, θ_em_, o_ec_ and ω_ec_

## Results

### Descriptive findings and bivariate associations

**Figure 3:**
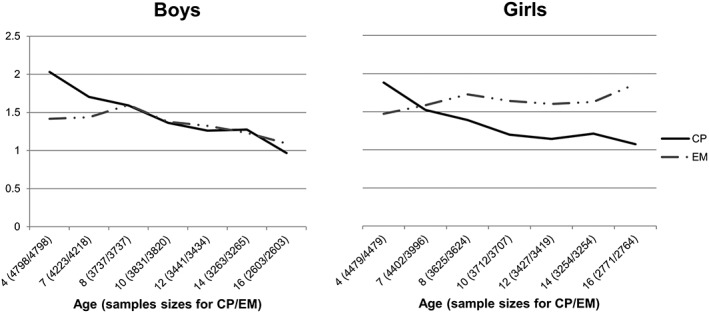
Observed mean levels of conduct (CP) and emotional (EM) problems by gender. Sample sizes for both observed measures at each time point are also displayed.

As expected, boys had higher mean levels of conduct problems, and girls higher scores for emotional difficulties, at all ages from 4 to 16 years (Figure 3 – where sample sizes for both measures at each time point are also displayed). There was also a significant gender difference in mean depression scores at age 18 (boys: mean [*M*] = 2.36, SD = 3.36, *n* = 1863; girls: *M* = 3.91, SD = 4.27, *n* = 2369; *t* (4230) = −12.83, *p* < 0.001).

Indicators of childhood conduct and emotional problems were positively correlated both within and across measurement waves; they were also correlated with measures of pre‐ and post‐natal maternal depression, and with young adult depression at age 18 (see Supporting Information Tables S1 and S2 – where sample sizes with complete data on the pairwise comparisons are also displayed).

### SEMs and path tracing (*n* = 6917 for boys and *n* = 6456 for girls)

#### Model 1: Is maternal depression associated with offspring depression at age 18?

Standardized regression coefficients from the first SEM (see Supporting Information, Figure S1) showed a significant positive association between pre‐/post‐natal maternal depression and young adult depression. A one SD increase in the latent factor of maternal depression was associated with average increases of 0.15 (95% CI = 0.09–0.20) and 0.16 (95% CI = 0.12–0.21) SDs in young adult depression scores for boys and girls, respectively. Acceptable fit was achieved in the models for both genders (boys: CFI = 0.97, RMSEA = 0.12 [90% CI = 0.11–0.14]; girls: CFI = 0.96, RMSEA = 0.13 [90% CI = 0.12–0.14]).

#### Model 2: Do childhood conduct problem trajectories mediate the effects of maternal depression on young adult depression?

Model 2 (Figure 1) tested the extent to which developmental trajectories of childhood conduct problems mediated these associations. Table 1 contains the total, direct, total indirect and time‐specific indirect effects of maternal depression on young adult depression derived from path tracing in Model 2. The total indirect effect of the conduct problem trajectories was significant for girls, but not for boys (see (*α*_cp_ × *β*_cp_) + (*γ*_cp_ × *δ*_cp_) + (*ε*_cp_ × *ζ*_cp_) in Model 2). Examination of the time‐specific effects showed that mediation was confined to initial (intercept, age four) problem levels for both girls and boys (see *α*_cp_ × *β*_cp_ in Model 2), and that there were no significant effects of changes in later levels of conduct problems (slopes) in either childhood or adolescence.

#### Model 3: Are trajectories of childhood emotional problems additional mediators?

Including emotional problem trajectories as additional mediators (Model 3 in Table 1, and Figure 2) diminished the effects of maternal depression on young adult depression, and also impacted estimates of other parameters. In this more comprehensive model only the indirect effects of conduct problem trajectories remained for girls, while for boys no mediation was suggested via either conduct or emotional difficulties (the total effect did not differ from the direct effect, and 95% Boostrap CIs overlapped, Model 3).

#### Model 4: Direct and indirect effects from reduced model

Finally we computed a reduced version of Model 3, with all its non‐significant paths constrained to zero. Table 2 shows the time‐specific indirect, total indirect, direct and total effects of maternal depression on young adult depression from this reduced model (Model 4, see Supporting Information Figures S2 and S3). For boys, no mediation via either conduct or emotional problems was suggested: none of the indirect effects were significant, and the total and direct effects appeared not to differ (95% Boostrap CIs overlapped). For girls, conduct problems at age four were again suggested as mediators of the maternal depression‐young adult depression association (see *α*_cp”_ × *β*_cp”_ in Table 2). In addition, this reduced model highlighted a pathway for girls linking maternal depression to larger increases (for those with initially low scores) or smaller decreases (for those with initially high scores) in emotional problems between ages 4–10 years and 10–16 years, and thence to higher levels of depression at age 18 (see *γ*_em”_ × *δ*_em”_ and *ε*_em”_ × *ζ*_em”_ in Table 2). A negative time‐specific indirect effect [also known as inconsistent (MacKinnon *et al*., 2007) or competitive mediation (Zhao *et al*., 2010)] was also suggested for girls (see *γ*_em”_ × *k*_em”_ × *ζ*_em”_ in Table 2).

## Discussion

We set out to examine the extent to which childhood conduct and emotional problems – well‐established sequelae of exposure to maternal depression – might also mediate risk for depression in offspring later in development. Our study benefited from established temporal relations between the chosen contemporaneous measures of pre‐/post‐natal maternal exposures and repeated measures of our chosen mediators in a large population‐based sample. In response to calls for life course studies to embrace methodologies that reflect the life course framework in ways that can move the prevention agenda forward (Wang, 2006), we employed a novel SEM approach to test hypothesized interrelations and estimate parameters representing hypothesized developmental processes (Ferrer and McArdle, 2010). Specifically, we initially used univariate piecewise latent trajectories of child conduct and emotional difficulties from ages 4–16 years to model changing levels of child/adolescent difficulties across development, and then examined these two sets of trajectories simultaneously as putative mediators in joint models. Such models can deal with several outcomes simultaneously. To our knowledge, this methodological application is novel in mediation with longitudinal data (von Soest and Hagtvetb, 2011). We consider it to have considerable potential in exploratory longitudinal mediation studies, adding increased power and accuracy through the use of latent intercepts and latent growth factors; it is likely to be especially valuable in developmental psychopathology, where multiple interrelated mediating factors can be expected to unfold over time, and to vary across developmental periods. Such modelling treats change as a unitary continuous process over time (Curran *et al*., 1996), allowing simultaneous evaluation of change at different developmental stages (Cheong *et al*., 2003) – here, childhood and adolescence – and enabling identification of time‐specific indirect effects (Khoo, 2001). The correlations/covariances between the growth rates factors as well as between the growth rates factors and intercepts provide useful information too for the trajectories of conduct problems and their co‐development with emotional problems (see Supporting Information for further details). None of these issues can be addressed through standard data tables from regression analyses, which would offer woefully unsatisfying data descriptions of the types of processes hypothesized here. For instance if all types of exposures (i.e. background such as maternal depression and intermediate such as conduct and emotional problems in the current study) were included as explanatory variables in the same model for the distal outcome of young adult depression at age 18, the resulting regression coefficients would measure mutually adjusted effects, that is, effects of background variables not mediated via the intermediate variables and effects of intermediate variables conditional on the background ones (De Stavola *et al*., 2006). In addition, as repeated measures of the same variables are taken over time, different interpretations would be possible depending on the parametrization of the conditioning variables.

In relation to model selection, we attempted to find models favoured by several criteria, to highlight the “best” of the candidate models. In our analyses BIC indicated Model 2 as the best fit to the data across all models, and Model 4 a better fit compared to Model 3. This was to be expected, as BIC generally favours “simpler models” (Kuha, 2004). However, likelihood ratio tests between nested models (here, Models 2 and 3) yielded *p*‐values < 0.001, and thus indicated a significantly better fit for Model 3 for both genders. Similar comparisons between Models 3 and 4 indicated the latter as the best fit for boys (*p*‐value < 0.001) but not for girls (*p*‐value = 0.557). We conclude that such differing estimates at least suggest bounds for the range of acceptable models (Kuha, 2004). We thus base our inference highlighting constant and changing effects across these different gender‐specific models.

In terms of substantive findings our analyses suggested that for girls, conduct problem levels very early in development (at age four years) were consistently indicated as mediators of effects of pre‐/early post‐natal depression in mothers on risk of depression in early adulthood (Models 2–4, Tables 1 and 2); later changes in levels of conduct problem showed no comparable effects. Interestingly, Nilsen *et al*. (2013) using a conventional modelling approach also identified links between maternal distress, early childhood behaviour problems and subsequent risk for offspring depression, and the most consistent evidence for links with depression in studies using latent growth curve analyses emerges in groups with early onset and persistent conduct problems. There was, however, some evidence for a pathway via changing levels of emotional difficulties, with larger increases (for those with initially low problems levels) or smaller decreases (for those with high initial levels) in emotional problems both in childhood (at ages 4–10 years) and adolescence (10–16 years). For boys, by contrast, the differing models provided less consistent findings. In particular, although a very small time‐specific indirect effect (0.027) via conduct problems at age four was initially indicated (Model 2, Table 1), this was no longer significant when emotional problems were included (Model 3), and so was not included in our final reduced model (Model 4). We take this to reflect redundancy among predictors of adolescent depression; early childhood conduct problems may indeed, however, be salient mediators of maternal depression, and future studies should continue to examine them alongside alternative additional mediators in boys.

Our findings need to be considered in light of some limitations. Bias due to measurement error could still occur in the selected measures, and there may be some bias due to attrition and violation of the Missing at Random Assumption. For more definitive conclusions about causality, further research is needed; in particular, it would be important to demonstrate that the associations we have documented are replicated in other ethnically similar samples and whether of course such results can be generalized to ethnically diverse samples. The linearity, normality distributional, and no unmeasured confounding or “ignorability” assumptions are made for all variables on the path diagrams, across the entire SEM (VanderWeele, 2012). Our SEM approach also assumed that observed repeated measures of maternal depression were independent of one another, being only related to each other through their common relationship with the latent variable representing cumulative exposure to pre‐natal and post‐natal depression.

Marginal structural modelling, a class of causal models (Hernán *et al*., 2000; Robins *et al*., 2000) – relatively new to the psychology literature – (VanderWeele *et al*., 2011), assessing effects of time‐varying exposures to maternal depression, could constitute alternative methods for the analysis of the current data. It is important to note that when the variables that confound the relationship between the exposure and the outcome also change with time, analyses based on standard linear regression or growth curve modelling will generally give biased estimates for time varying exposures because they cannot appropriately adjust for confounding variables that change over time and may also be affected by prior treatment/exposure (Daniel *et al*., 2013; Hernán *et al*., 2002; Robins *et al*., 2000). At this stage the current causal inference literature does not allow for exposures and mediators themselves to vary over time, and an approach that fully accommodates time‐varying exposures and mediators and time‐varying confounding is still under development (VanderWeele and Tchetgen, 2014). Very little work currently exists in this specific causal modelling literature for longitudinal data with time varying exposures and mediators – as is the case in our study (Daniel *et al*., 2015; van der Laan and Petersen, 2008; VanderWeele, 2009). Thus our SEM approach still provides suggestive results – generating valid hypotheses for future research which could embrace such modern methodological tools. For instance, another study (Pearson *et al*., 2013) although it did not directly test the mechanisms of the transmission of depression from mother to adolescent suggests that that study's findings provide indirect evidence that the pathways from ante‐natal depression and post‐natal depression are different. Since the scope of our study did not include differentiation between exposure to ante‐natal and post‐natal maternal depression, to elucidate these pathways further, we thus propose future studies to fit marginal structural models in the ALSPAC dataset – considering the current suggested mediators, gender as a moderator and adjusting for relevant time varying confounders

In conclusion, our study provides evidence that in boys, early childhood conduct problems may be a mediator of the intergenerational transmission of depression, and that future research on these associations including alternative mediators and confounders would be warranted. For girls, our findings support the hypothesis that both future research and intervention and prevention strategies should focus on conduct problems early in childhood and emotional difficulties across childhood and adolescence, as mediators of depression risk. The realization that conduct problems may underlie the transition to depression is crucial clinically and in public health terms. There is a solid evidence base for the treatment of conduct problems in young people and for the feasibility of applying such treatments to non‐clinical settings (Pilling *et al*., 2013). Clinicians should be aware that apart from alleviating concurrent impairment due to conduct problems and reducing the probability of transition to future antisocial behaviours (Scott *et al*., 2014), such treatments may reduce the chances of transitioning to depression. Understanding the mechanisms of such transitions could help further refine such treatments.

## Declaration of interest statement

The authors have no competing interests.

## Supporting information


**Figure S1.** Path diagram for Model 1
**Table S1.** Zero Order Spearman Correlations for all Exposure, Mediator and Dependent Variables for Boys
**Table S2.** Zero Order Spearman Correlations for all Exposure, Mediator and Dependent Variables for Girls
**Table S3.** Standardized regression estimates and (95 % Confidence Intervals (CIs)) from Models 2 and 3
**Table S4** Standardized regression estimates and (95 % Confidence Intervals (CIs)) from Model 4
**Figure S2,** Path diagram for Model 4 for Boys
**Figure S3,** Path diagram for Model 4 for Girls

Supporting info item
