# Supplementary material for: Pathways from maternal depression to young adult offspring depression: an exploratory longitudinal mediation analysis
Source: Int J Methods Psychiatr Res. 2016 Jul 29;26(2):e1520. doi: 10.1002/mpr.1520 (PMC5484332; doi:10.1002/mpr.1520)
Supplement: Supplementary file 1 — Supporting info item [file MPR-26-e1520-s001.docx]

**Supplementary material**

accompanying manuscript:

**Pathways from maternal depression to young adult offspring depression: an exploratory longitudinal mediation analysis**

*Contents*

e-Figure 1. Path diagram for Model 1……………………………………………………………………………………………..p.2

e-Table 1. Zero Order Spearman Correlations for all Exposure, Mediator and Dependent Variables for Boys………………….p.3-4

e-Table 2. Zero Order Spearman Correlations for all Exposure, Mediator and Dependent Variables for Girls…………………..p.5-6

e-Table 3. Standardized regression estimates and (95 % Confidence Intervals (CIs)) from Models 2 and 3…………………….p.9-11

e-Table 4 Standardized regression estimates and (95 % Confidence Intervals (CIs)) from Model 4………………………………p.13-14

e-Figure 2, Path diagram for Model 4 for Boys……………………………………………………………………………………p.15

e-Figure 3, Path diagram for Model 4 for Girls…………………………………………………………………………………….p.16

**e-Figure 1**. Path diagram for Model 1

YA Depression

18 years

EPDS

12 weeks

antenatally

EPDS

32 weeks

antenatally

EPDS

8 weeks

post-natally

EPDS

8 months

post-natally

c

λ2

λ1

λ3

λ4

*Descriptive Measures*

**e Table 1**. Zero Order Spearman Correlations for all Exposure, Mediator and Dependent Variables for Boys‡

| Variable | 1 | 2 | 3 | 4 | 5 | 6 | 7 | 8 | 9 | 10 | 11 | 12 | 13 | 14 | 15 | 16 | 17 | 18 | 19 |
| --- | --- | --- | --- | --- | --- | --- | --- | --- | --- | --- | --- | --- | --- | --- | --- | --- | --- | --- | --- |
| 1. Conduct problems at age 4 |  | 0.484  (3912) | 0.485  (3471) | 0.435  (3497) | 0.357  (3172) | 0.356  (3031) | 0.249  (2424) | 0.220  (4798) | 0.170  (3909) | 0.195  (3471) | 0.177  (3489) | 0.166  (3166) | 0.192  (3034) | 0.119  (2422) | 0.146  (4367) | 0.159  (4503) | 0.153  (4559) | 0.172  (4511) | 0.073  (1665) |
| 2. Conduct problems at age 7 |  |  | 0.598  (3380) | 0.562  (3422) | 0.501  (3092) | 0.464  (2949) | 0.348  (2372) | 0.158  (3912) | 0.248  (4214) | 0.217  (3380) | 0.221  (3413) | 0.242  (3086) | 0.227  (2952) | 0.187  (2371) | 0.156  (3864) | 0.165  (3995) | 0.171  (4030) | 0.189  (3998) | 0.035  (1612) |
| 3. Conduct problems at age 8 |  |  |  | 0.610  (3250) | 0.549  (2964) | 0.510  (2816) | 0.372  (2292) | 0.161  (3471) | 0.186  (3378) | 0.263  (3737) | 0.212  (3243) | 0.230  (2956) | 0.247  (2818) | 0.161  (2291) | 0.154  (3421) | 0.152  (3527) | 0.159  (3571) | 0.174  (3529) | 0.027  (1537) |
| 4. Conduct problems at age 10 |  |  |  |  | 0.576  (3100) | 0.546  (2941) | 0.398  (2358) | 0.140  (3497) | 0.175  (3417) | 0.190  (3250) | 0.249  (3815) | 0.216  (3093) | 0.226  (2943) | 0.174  (2356) | 0.141  (3512) | 0.157  (3615) | 0.148  (3638) | 0.158  (3606) | 0.016  (1591) |
| 5. Conduct problems at age 12 |  |  |  |  |  | 0.600  (2873) | 0.444  (2320) | 0.122  (3172) | 0.162  (3090) | 0.197  (2964) | 0.219  (3093) | 0.277  (3425) | 0.233  (2873) | 0.180  (2318) | 0.113  (3174) | 0.125  (3268) | 0.116  (3280) | 0.127  (3245) | 0.046  (1544) |
| 6. Conduct problems at age 13 |  |  |  |  |  |  | 0.472  (2317) | 0.112  (3031) | 0.150  (2948) | 0.185  (2816) | 0.182  (2932) | 0.209  (2866) | 0.273  (3259) | 0.208  (2318) | 0.158  (3015) | 0.169  (3097) | 0.133  (3120) | 0.155  (3079) | 0.056  (1519) |
| 7. Conduct problems at age 16 |  |  |  |  |  |  |  | 0.045  (2424) | 0.112  (2371) | 0.101  (2292) | 0.118  (2352) | 0.178  (2320) | 0.169  (2319) | 0.244  (2594) | 0.130  (2430) | 0.130  (2479) | 0.111  (2504) | 0.129  (2480) | 0.072  (1419) |
| 8. Emotional problems at age 4 |  |  |  |  |  |  |  |  | 0.417  (3909) | 0.391  (3471) | 0.310  (3489) | 0.286  (3166) | 0.250  (3034) | 0.201  (2422) | 0.161  (4367) | 0.155  (4503) | 0.165  (4559) | 0.172  (4511) | 0.064  (1665) |
| 9. Emotional problems at age 7 |  |  |  |  |  |  |  |  |  | 0.532  (3378) | 0.475  (3408) | 0.432  (3084) | 0.387  (2951) | 0.305  (2370) | 0.177  (3860) | 0.157  (3992) | 0.193  (4029) | 0.198  (3997) | 0.100  (1611) |
| 10. Emotional problems at age 8 |  |  |  |  |  |  |  |  |  |  | 0.528  (3243) | 0.473  (2956) | 0.437  (2818) | 0.333  (2291) | 0.191  (3421) | 0.184  (3527) | 0.198  (3571) | 0.229  (3529) | 0.131  (1537) |
| 11. Emotional problems at age 10 |  |  |  |  |  |  |  |  |  |  |  | 0.518  (3087) | 0.473  (2935) | 0.335  (2351) | 0.207  (3501) | 0.209  (3606) | 0.204  (3626) | 0.204  (3597) | 0.151  (1588) |
| 12. Emotional problems at age 12 |  |  |  |  |  |  |  |  |  |  |  |  | 0.547  (2869) | 0.403  (2318) | 0.214  (3170) | 0.208  (3262) | 0.213  (3272) | 0.199  (3241) | 0.135  (1546) |
| 13. Emotional problems at age 13 |  |  |  |  |  |  |  |  |  |  |  |  |  | 0.432  (2320) | 0.174  (3017) | 0.179  (3099) | 0.201  (3122) | 0.190  (3081) | 0.161  (1522) |
| 14. Emotional problems at age 16 |  |  |  |  |  |  |  |  |  |  |  |  |  |  | 0.164  (2428) | 0.158  (2477) | 0.190  (2504) | 0.174  (2480) | 0.216  (1423) |
| 15. EPDS at 18 weeks antenatally |  |  |  |  |  |  |  |  |  |  |  |  |  |  |  | 0.638  (5591) | 0.536  (5299) | 0.512  (5106) | 0.113  (1717) |
| 16. EPDS at 32 weeks antenatally |  |  |  |  |  |  |  |  |  |  |  |  |  |  |  |  | 0.579  (5504) | 0.550  (5299) | 0.081  (1756) |
| 17. EPDS at 8 weeks post-natally |  |  |  |  |  |  |  |  |  |  |  |  |  |  |  |  |  | 0.612  (5383) | 0.110  (1751) |
| 18. EPDS at 8 months post-natally |  |  |  |  |  |  |  |  |  |  |  |  |  |  |  |  |  |  | 0.091  (1746) |
| 19 Young Adult Depression at age 18 |  |  |  |  |  |  |  |  |  |  |  |  |  |  |  |  |  |  |  |

‡ Displayed correlations are based on sample sizes given in brackets (the sample sizes are from complete data on the displayed pairwise comparisons)

**e Table 2**. Zero Order Spearman Correlations for all Exposure, Mediator and Dependent Variables for Girls◊

| Variable | 1 | 2 | 3 | 4 | 5 | 6 | 7 | 8 | 9 | 10 | 11 | 12 | 13 | 14 | 15 | 16 | 17 | 18 | 19 |
| --- | --- | --- | --- | --- | --- | --- | --- | --- | --- | --- | --- | --- | --- | --- | --- | --- | --- | --- | --- |
| 1. Conduct problems at age 4 |  | 0.461  (3345) | 0.460  (3380) | 0.408  (3135) | 0.359  (2976) | 0.368  (2528) | 0.272  (4479) | 0.222  (3691) | 0.156  (3343) | 0.192  (3377) | 0.174  (3130) | 0.174  (2976) | 0.190  (2522) | 0.166  (4082) | 0.156  (4228) | 0.159  (4252) | 0.170  (4230) | 0.177  (2042) | 0.097  (3697) |
| 2. Conduct problems at age 7 |  |  | 0.590  (3302) | 0.541  (3318) | 0.466  (3060) | 0.447  (2897) | 0.331  (2481) | 0.141  (3697) | 0.259  (3996) | 0.214  (3302) | 0.225  (3314) | 0.220  (3054) | 0.196  (2896) | 0.192  (2475) | 0.147  (3661) | 0.127  (3799) | 0.130  (3835) | 0.166  (3802) | 0.100  (4002) |
| 3. Conduct problems at age 8 |  |  |  | 0.589  (3207) | 0.511  (2953) | 0.488  (2804) | 0.390  (2433) | 0.147  (3345) | 0.207  (3300) | 0.266  (3622) | 0.221  (3203) | 0.226  (2951) | 0.189  (2806) | 0.200  (2428) | 0.137  (3323) | 0.143  (3461) | 0.144  (3490) | 0.171  (3458) | 0.089  (1904) |
| 4. Conduct problems at age 10 |  |  |  |  | 0.570  (3079) | 0.547  (2897) | 0.393  (2513) | 0.100  (3380) | 0.171  (3313) | 0.183  (3206) | 0.246  (3702) | 0.215  (3072) | 0.192  (2897) | 0.190  (2509) | 0.129  (3401) | 0.143  (3534) | 0.126  (3559) | 0.144  (3521) | 0.095  (1982) |
| 5. Conduct problems at age 12 |  |  |  |  |  | 0.583  (2882) | 0.439  (2454) | 0.108  (3135) | 0.170  (3058) | 0.191  (2951) | 0.200  (3075) | 0.270  (3416) | 0.232  (2881) | 0.223  (2450) | 0.147  (3130) | 0.167  (3252) | 0.163  (3283) | 0.167  (3255) | 0.147  (1923) |
| 6. Conduct problems at age 13 |  |  |  |  |  |  | 0.477  (2427) | 0.089  (2976) | 0.190  (2894) | 0.170  (2804) | 0.191  (2897) | 0.231  (2877) | 0.308  (3250) | 0.252  (2420) | 0.157  (2986) | 0.154  (3093) | 0.155  (3111) | 0.166  (3074) | 0.145  (1859) |
| 7. Conduct problems at age 16 |  |  |  |  |  |  |  | 0.080  (2528) | 0.137  (2477) | 0.154  (2432) | 0.157  (2511) | 0.189  (2451) | 0.200  (2426) | 0.299  (2763) | 0.175  (2539) | 0.157  (2650) | 0.149  (2665) | 0.153  (2650) | 0.134  (1778) |
| 8. Emotional problems at age 4 |  |  |  |  |  |  |  |  | 0.415  (3691) | 0.387  (3343) | 0.341  (3377) | 0.306  (3130) | 0.254  (2976) | 0.201  (2522) | 0.136  (4082) | 0.149  (4228) | 0.171  (4252) | 0.166  (4230) | 0.047  (2042) |
| 9. Emotional problems at age 7 |  |  |  |  |  |  |  |  |  | 0.555  (3299) | 0.504  (3311) | 0.435  (3051) | 0.414  (2894) | 0.336  (2471) | 0.164  (3655) | 0.170  (3793) | 0.169  (3831) | 0.195  (3796) | 0.103  (1983) |
| 10. Emotional problems at age 8 |  |  |  |  |  |  |  |  |  |  | 0.525  (3201) | 0.464  (2949) | 0.432  (2806) | 0.381  (2427) | 0.185  (3323) | 0.191  (3461) | 0.204  (3488) | 0.213  (3457) | 0.127  (1904) |
| 11. Emotional problems at age 10 |  |  |  |  |  |  |  |  |  |  |  | 0.518  (3069) | 0.484  (2897) | 0.388  (2507) | 0.175  (3397) | 0.167  (3529) | 0.188  (3553) | 0.205  (3516) | 0.111  (1981) |
| 12. Emotional problems at age 12 |  |  |  |  |  |  |  |  |  |  |  |  | 0.570  (2877) | 0.448  (2449) | 0.178  (3124) | 0.191  (3245) | 0.194  (3274) | 0.178  (3249) | 0.153  (1924) |
| 13. Emotional problems at age 13 |  |  |  |  |  |  |  |  |  |  |  |  |  |  |  |  |  |  |  |
| 14. Emotional problems at age 16 |  |  |  |  |  |  |  |  |  |  |  |  |  | 0.504  (2419) | 0.171  (2987) | 0.191  (3094) | 0.189  (3112) | 0.182  (3074) | 0.180  (1859) |
| 15. EPDS at 18 weeks antenatally |  |  |  |  |  |  |  |  |  |  |  |  |  |  | 0.192  (2533) | 0.203  (2643) | 0.178  (2658) | 0.191  (2645) | 0.220  (1777) |
| 16. EPDS at 32 weeks antenatally |  |  |  |  |  |  |  |  |  |  |  |  |  |  |  | 0.629  (5304) | 0.524  (5013) | 0.499  (4812) | 0.097  (2151) |
| 17. EPDS at 8 weeks post-natally |  |  |  |  |  |  |  |  |  |  |  |  |  |  |  |  | 0.567  (5208) | 0.536  (5013) | 0.108  (2248) |
| 18. EPDS at 8 months post-natally |  |  |  |  |  |  |  |  |  |  |  |  |  |  |  |  |  | 0.606  (5053) | 0.110  (2242) |
| 19 Young Adult Depression at age 18 |  |  |  |  |  |  |  |  |  |  |  |  |  |  |  |  |  |  |  |

◊Displayed correlations are based on sample sizes given in brackets (the sample sizes are from complete data on the displayed pairwise comparisons)

*Standardized regression coefficients from Models 2 and 3*

Table 3 shows coefficients from fitting Models 2 and 3. Model 2 (testing for mediation of these associations via child/adolescent conduct problems) suggested partial mediation of these effects in both genders. Specifically, maternal depression significantly predicted higher mean levels of conduct problems at age 4 (i.e. in the intercept, see αcp), and mean levels of early childhood conduct problems were in turn significantly associated with mean levels of young adult depression (see βcp). Changes in levels of conduct problems across childhood (slope 1, ages 4-10 years) were only associated with later depression risk for girls (see δcp), whereas changes in early adolescence (slope 2, ages 10-16 years) were associated with later depression risk in both genders: larger early adolescent increases in conduct problems (for those with initially low scores) or smaller decreases (for those with initially high scores) were positively associated with higher mean levels of depression at age 18 (see ζcp). Maternal depression was unrelated to childhood changes in levels of conduct problems (ages 4-10 years, γcp). In boys maternal depression predicted significantly lower mean changes in conduct problem levels across the early adolescent years (ages 10-16), while in girls this association was significant and positive (see εcp).

Adding the trajectories of emotional problems (Model 3), led to attenuation of the effect of initial (age 4) mean levels of conduct problems on young adult depression for girls, and reduced the effect for boys to non-significance (see βcp’). For both genders, this model also suggested a stronger effect of maternal depression on initial (age 4) mean levels of childhood emotional problems than on early childhood conduct problems (see αcp’ and αem). Age 4 emotional difficulties were not related to risk for early adult depression, but subsequent changes in levels of emotional problems were: larger increases (or smaller decreases) in emotional problems between both ages 4-10 and 10-16 years were positively associated with young adult depression (see δem and ζem). For girls because the relevant ratios of the regression coefficients γ_em_, δ_em_, ε_em_ and ζ_em_ and their corresponding standard errors were small i.e. less than 6 –this led for the time-specific indirect effects of changes in emotional problems between ages 4-10 and 10-16 years for girls not to be significant (see γ_em_ * δ_em_ and ε_em_* ζ_em_ in Model 3 in Table 1 in thearticle).

Some effects for boys were changed when compared from Model 2 to Model 3: in the latter maternal depression predicted significantly higher mean changes in conduct problems during the ages 4-10 but not during the ages 10-16 (see γcp’ and εcp’); mean changes in conduct problems during the ages 4-10 were negatively associated with young adult depression (see δcp’).

*Trajectories of conduct problems from Model 2*

The negative covariance between estimates for the intercept and slope of conduct problems in Model 2 indicated that individuals with high age 4 conduct problem scores increased less (or decreased more) from ages 4-10 years than individuals with lower age 4 scores (see Cov_cp_ints1). In a similar way the negative covariance in this same model between the two slopes implied that on average individuals with larger changes prior to the childhood-adolescent transition (age 10) were likely to experience smaller changes following the transition (see Cov_cp_s1s2).

*Trajectories of conduct and emotional problems and their co-development from Models 3 and 4*

Finally, Model 3 confirmed that initial mean levels as well as changes in levels of conduct and emotional problems were positively correlated (Cov_cp_em__int, Cov_cp_em__s1 and Cov_cp_em__s2). Such findings clearly indicate a pattern of parallel growth or stability for conduct and emotional problems that corresponds to large, positive correlations among the intercept and growth rate factors. These relationships remained the same in Model 4, Table 4. Furthermore, Model 4 indicated zero covariance between the first slope and initial status for emotional problems; the interpretation of such a finding is that where one starts at age 4 has no bearing on whether one increases or decreases between ages 4-10 for emotional problems.

**e Table 3**. Standardized regression estimates and (95 % Confidence Intervals (CIs)) from Models 2 and 3

| **Model 2◦◦**  **Conduct problem trajectories as mediators** | | | | **Model 3◦◦◦**  **Conduct and emotional problem trajectories as mediators** | | | | |  |  |  |  |  |  |  |  |
| --- | --- | --- | --- | --- | --- | --- | --- | --- | --- | --- | --- | --- | --- | --- | --- | --- |
| Parameters | | Standardized Estimates  (95 % CIs) | | Parameters | Standardized Estimates  (95 % CIs) | | | |  |  |  |  |  |  |  |  |
|  | | Boys  (n=6917) | Girls  (n=6456) |  | Boys  (n=6917) | Girls  (n=6456) | | |  |  |  |  |  |  |  |  |
| c’:  Mat dep YA Depression 18 | | 0.128  (0.072 to 0.184)  p < 0.001 | 0.095  (0.045 to 0.144)  p < 0.001 | c’’:  Mat dep YA Depression 18 | 0.110  (0.041 to 0.179)  p = 0.002 | 0.092  (0.039 to 0.145)  p = 0.001 | | |  |  |  |  |  |  |  |  |
| αcp:  Mat dep Int_cp | | 0.293  (0.256 to 0.330)  p < 0.001 | 0.304  (0.266 to 0.342)  p < 0.001 | αcp’:  Mat dep Int_cp | 0.296  (0.258 to 0.333)  p < 0.001 | 0.306  (0.267 to 0.344)  p < 0.001 | | |  |  |  |  |  |  |  |  |
| βcp:  Int_cp YA Depression 18 | | 0.093  (0.029 to 0.156)  p = 0.004 | 0.206  (0.148 to 0.263)  p < 0.001 | βcp’:  Int_cp YA Depression 18 | 0.044  (-0.057 to 0.145)  p = 0.394 | 0.123  (0.054 to 0.193)  p = 0.001 | | |  |  |  |  |  |  |  |  |
| γcp:  Mat dep S1_cp | | 0.018  (-0.032 to 0.069)  p = 0.476 | -0.029  (-0.080 to 0.022)  p = 0.269 | γcp’:  Mat dep S1_cp | 0.126  (0.061 to 0.192)  p < 0.001 | 0.052  (-0.006 to 0.111)  p = 0.078 | | |  |  |  |  |  |  |  |  |
| δcp:  S1_cp YA Depression 18 | | 0.014  (-0.069 to 0.098)  p = 0.739 | 0.189  (0.110 to 0.268)  p < 0.001 | δcp’:  S1_cp YA Depression 18 | -0.360  (-0.679 to -0.042)  p = 0.026 | 0.039  (-0.081 to 0.159)  p = 0.527 | | |  |  |  |  |  |  |  |  |
| εcp:  Mat_dep S2_cp | | -0.084  (-0.146 to -0.022)  p = 0.008 | 0.069  (0.008 to 0.131)  p = 0.027 | εcp’:  Mat_dep S2_cp | -0.051  (-0.117 to 0.014)  p = 0.125 | 0.099  (0.036 to 0.162)  p = 0.002 | | |  |  |  |  |  |  |  |  |
| ζcp:  S2_cp YA Depression 18 | | 0.099  (0.003 to 0.196)  p = 0.043 | 0.240  (0.155 to 0.326)  p < 0.001 | ζcp’:  S2_cp YA Depression 18 | -0.165  (-0.365 to 0.036)  p = 0.107 | 0.088  (-0.075 to 0.250)  p = 0.289 | | |  |  |  |  |  |  |  |  |
| Cov_cp_ints1 | | -0.312  (-0.367 to -0.257)  p < 0.001 | -0.411  (-0.460 to -0.363)  p < 0.001 | Cov_cp_ints1 | -0.168  (-0.254 to -0.081)  p < 0.001 | -0.320  (-0.381 to -0.259)  p < 0.001 | | |  |  | | | | | |  |
| Cov_cp_s1s2 | | -0.490  (-0.546 to -0.434)  p < 0.001 | -0.502  (-0.553 to -0.452)  p < 0.001 | Cov_cp_s1s2 | -0.391  (-0.468 to -0.314)  p < 0.001 | -0.427  (-0.488 to -0.367)  p < 0.001 | |  |  |  | |  |  |  |  |  |
|  | |  |  | αem:  Mat dep Int_em | 0.395  (0.344 to 0.447)  p < 0.001 | 0.357  (0.307 to 0.406)  p < 0.001 | |  |  |  | |  |  |  |  |  |
|  | |  |  | βem:  Int_em YA Depression 18 | -0.032  (-0.269 to 0.206)  p = 0.794 | 0.022  (-0.066 to 0.110)  p = 0.625 | |  |  |  | |  |  |  |  |  |
|  | |  |  | γem:  Mat dep S1_em | 0.116  (-0.001 to 0.234)  p = 0.053 | 0.096  (0.015 to 0.176)  p = 0.020 | |  |  |  | |  |  |  |  |  |
|  | |  |  | δem:  S1_em YA Depression 18 | 0.591  (0.252 to 0.930)  p = 0.001 | 0.243  (0.136 to 0.351)  p < 0.001 | |  |  |  | |  |  |  |  |  |
|  | |  |  | εem:  Mat_dep S2_em | -0.006  (-0.068 to 0.056)  p = 0.852 | 0.072  (0.018 to 0.125)  p = 0.008 | |  |  |  | |  |  |  |  |  |
|  | |  |  | ζem:  S2_em YA Depression 18 | 0.480  (0.253 to 0.707)  p < 0.001 | 0.236  (0.084 to 0.388)  p = 0.002 | |  |  |  | |  |  |  |  |  |
|  | |  |  | θem† :  i_em S1_em | 0.225  (-0.245 to 0.695)  p = 0.348 | 0.006  (-0.222 to 0.235)  p = 0.956 |  | | | |  | |  |  |  |  |
|  | |  |  | κem††:  S1_em S2_em | -0.759  (-0.833 to -0.684)  p < 0.001 | -0.525  (-0.598 to -0.451)  p < 0.001 |  | |  |  | |  |  |  |  |  |
|  | |  |  | Cov_cp_em__int | 0.501  (0.437 to 0.565)  p < 0.001 | 0.414  (0.352 to 0.477)  p < 0.001 |  | |  |  | |  |  |  |  |  |
|  | |  |  | ξec:  i_em S1_cp | -0.266  (-0.354 -0.177)  p < 0.001 | -0.238  (-0.309 to -0.168)  p < 0.001 |  | |  |  | |  |  |  |  |  |
|  | |  |  | οec:  i_cp S1_em | -0.144  (-0.401 to 0.114)  p = 0.274 | 0.015  (-0.104 to 0.134)  p = 0.804 |  | |  |  | |  |  |  |  |  |
|  | |  |  | Cov_cp_em__s1 | 0.512  (0.309 to 0.715)  p < 0.001 | 0.378  (0.305 to 0.451)  p < 0.001 |  | |  |  | |  |  |  |  |  |
|  | |  |  | πec:  S1_em S2_cp | -0.222  (-0.306 to -0.138)  p < 0.001 | -0.288  (-0.371 to -0.204)  p < 0.001 |  | |  |  | |  |  |  |  |  |
|  | |  |  | ω_ec_:  S1_cp S2_em | 0.292  (0.167 to 0.417)  p < 0.001 | 0.046  (-0.041 to 0.133)  p = 0.298 | | | | | | | |  |  |  |
|  |  |  |  | Cov_cp_em__s2 | 0.526  (0.441 to 0.611)  p < 0.001 | 0.577  (0.499 to 0.654)  p < 0.001 | | |  |  |  |  |  |  |  |  |

Abbreviations: Mat dep, Maternal depression; YA Depression 18, young adult depression at age of 18 as assessed by the Revised Clinical Interview Schedule; Int_cp, Intercept for conduct problems at age of 4; S1_cp, Slope 1 for conduct problems-change of conduct problems during ages 4-10; S2_cp, Slope 2 for conduct problems-change of condut problems during ages 10-16; Cov_cp_ints1, Covariance between intercept and slope 1 for conduct problems; Cov_cp_s1s2, Covariance between slope 1 and slope 2 for conduct problems; Int_em, Intercept for emotional problems at age of 4; S1_em, Slope 1 for emotional problems-change of emotional problems during ages 4-10; S2_em, Slope 2 for emotional problems-change of emotional problems during ages 10-16; Cov_cp_em__int, Covariance between intercepts for conduct and emotional problems; Cov_cp_em__s1, Covariance between slopes 1 for conduct and emotional problems; Cov_cp_em__s2, Covariance between slopes 2 for conduct and emotional problems.

◦◦,◦◦◦ Note: Models 2 and 3 are graphically presented in Figures 1 and 2 in the article respectively. In Model 2 we tested if the conduct problems and at which age they acted as mediators in the afore mentioned relationship between maternal depression and young adult depression and Model 3 is identical to Model 2 where in addition to the conduct problems we consider more mediators as represented by the trajectory of emotional problems in a similar fashion as for the conduct problems.

†, †† Note: Additional analyses (not represented here but available from the authors) showed considerable negative relationship between intercept and Slope 1 for emotional problems as well as negative relationship between Slopes 1 and 2 for emotional problems. Based on recommendations by von Soest and Hagtvetb we decided to control Slope 1 for emotional problems by its corresponding intercept (this is represented by regression coefficient θ_em_ in this Table and in Figure 2 in the article). We also controlled Slope 2 for emotional problems by its corresponding Slope 1 (this is represented by κ_em_ in this Table and Figure 2 in the article). Allowing for these specific adjustments, then effects like γ_em_ and ε_em_ are *net* effects in the sense that they are adjusted for differences in initial status of emotional problems and changes of emotional problems between ages 4-10 among those with more and less depressed mothers pre- and post-pregnancy. However, when we tried to incorporate in Model 3 at the same time control of Slope 1 for conduct problems by its corresponding intercept and Slope 2 for conduct problems by its corresponding Slope 1, we got messages for both boys’ and girls’ relevant data after the estimation process in Mplus outputs of negative residual variance for some latent variable and correlation between latent variables>1 which simply mean that such a model would not be appropriate for these data. This is why for conduct problems we estimate instead covariances of intercept with Slope 1 and covariances of Slope 1 with Slope 2 (i.e. Cov__cp_ints1_ & Cov__cp_s1s2_ in the above Table for Model 3 and in Figure 2 in the article) .

**e Table 4**. Standardized regression estimates and (95 % Confidence Intervals (CIs)) from Model 4

| Model 4† | |  | |
| --- | --- | --- | --- |
| Parameters | Standardized Estimates  (95 % CIs) | Parameters | Standardized Estimates  (95 % CIs) |
| Boys (n = 6917) | | Girls (n = 6456) | |
| c’’:  Mat dep YA Depression 18 | 0.149  (0.097 to 0.202)  p < 0.001 | c’’:  Mat dep YA Depression 18 | 0.102  (0.053 to 0.151)  p < 0.001 |
| αcp’:  Mat dep Int_cp | 0.300  (0.263 to 0.337)  p < 0.001 | αcp’:  Mat dep Int_cp | 0.322  (0.290 to 0.355)  p < 0.001 |
| γcp’:  Mat dep S1_cp | 0.079  (0.022 to 0.136)  p = 0.006 | βcp’:  Int_cp YA Depression 18 | 0.113  (0.060 to 0.166)  p < 0.001 |
| δcp’:  S1_cp YA Depression 18 | -0.222  (-0.319 to -0.124)  p < 0.001 | εcp’:  Mat_dep S2_cp | 0.122  (0.066 to 0.178)  p < 0.001 |
| Cov_cp_ints1 | -0.189  (-0.266 to -0.112)  p < 0.001 | Cov_cp_ints1 | -0.332  (-0.388 to -0.276)  p < 0.001 |
| Cov_cp_s1s2 | -0.392  (-0.463 to -0.321)  p < 0.001 | Cov_cp_s1s2 | -0.430  (-0.489 to -0.371)  p < 0.001 |
| αem:  Mat dep Int_em | 0.433  (0.398 to 0.467)  p < 0.001 | αem:  Mat dep Int_em | 0.351  (0.307 to 0.395)  p < 0.001 |
| δem:  S1_em YA Depression 18 | 0.465  (0.352 to 0.579)  p < 0.001 | γem:  Mat dep S1_em | 0.101  (0.040 to 0.162)  p = 0.001 |
| ζem:  S2_em YA Depression 18 | 0.322  (0.213 to 0.431)  p < 0.001 | δem:  S1_em YA Depression 18 | 0.274  (0.204 to 0.344)  p < 0.001 |
| κem††:  S1_em S2_em | -0.734  (-0.797 to -0.670)  p < 0.001 | εem:  Mat_dep S2_em | 0.069  (0.017 to 0.122)  p = 0.009 |
| Cov_cp_em__int | 0.465  (0.419 to 0.510)  p < 0.001 | ζem:  S2_em YA Depression 18 | 0.306  (0.233 to 0.379)  p < 0.001 |
| ξec:  i_em S1_cp | -0.257  (-0.337 to -0.176)  p < 0.001 | κem:  S1_em S2_em | -0.505  (-0.567 to -0.443)  p < 0.001 |
| Cov_cp_em__s1 | 0.440  (0.361 to 0.519)  p < 0.001 | Cov_cp_em__int | 0.405  (0.360 to 0.450)  p < 0.001 |
| πec:  S1_em S2_cp | -0.247  (-0.327 to -0.167)  p < 0.001 | ξec:  i_em S1_cp | -0.210  (-0.265 to -0.155)  p < 0.001 |
| ωec:  S1_cp S2_em | 0.249  (0.156 to 0.343)  p < 0.001 | Cov_cp_em__s1 | 0.371  (0.307 to 0.435)  p < 0.001 |
| Cov_cp_em__s2 | 0.508  (0.427 to 0.589)  p < 0.001 | πec:  S1_em S2_cp | -0.288  (-0.369 to -0.207)  p < 0.001 |
|  |  | Cov_cp_em__s2 | 0.589  (0.513 to 0.665)  p < 0.001 |

†Model 4 contains same pathways as Model 3 for each gender separately but fixing at 0 those which were not significant in the latter (i.e. in Model 3). For instance for boys, the pathways fixed at 0 in Model 4 were as follows: βcd’, εcd’,ζcd’, βem, γem, εem, θem and οec. For girls, the pathways fixed at 0 in Model 4 were as follows: γcp’, δcp’, ζcp’, βem, θem, οec and ωec.

**e Figure 2**. Path diagram for Model 4 for Boys

**e Figure 3**. Path diagram for Model 4 for Girls
